# Change in Patient MELD-Na and Albumin Level From the Time of Celiac Disease Diagnosis to Six Months Later After Gluten-Free Diet

**DOI:** 10.7759/cureus.8237

**Published:** 2020-05-22

**Authors:** Rajesh Essrani, Andrea Berger

**Affiliations:** 1 Internal Medicine, Geisinger Medical Center, Danville, USA; 2 Internal Medicine, Lehigh Valley Health Network, Allentown, USA; 3 Biostatistics, Geisinger Medical Center, Danville, USA

**Keywords:** cirrhosis, celiac disease, meld-na, albumin

## Abstract

Background & aims

Celiac disease (CD) is a multisystem disorder triggered by dietary gluten in genetically predisposed individuals that may affect any organ system, including the liver. We evaluated a change in patient model for end-stage liver disease (MELD)-Na and albumin level from the time of celiac disease diagnosis to six months later, after implementing a gluten-free diet.

Methods

A retrospective study was conducted from January 1, 2006, to June 30, 2018. CD was diagnosed based on celiac antibodies and/or histopathological data. MELD-Na and albumin were calculated at the start of the gluten-free diet and six months later. Additional variables like gender, ethnicity, serum IgA level, serum IgG level, human leukocyte antigen (HLA) type, and markers of end-stage liver disease were collected. Descriptive statistics, including means, were reported with the standard deviation for the continuous variables along with frequencies and percentages for all categorical variables.

Results

A total of 18 patients (55.6% male) were identified as having both cirrhosis and CD. The mean age at the time of celiac diagnosis was 53.6, and 94.4% were Caucasian. CD was diagnosed using celiac antibodies (100%) and histopathological data (44.4%). Most common celiac antibodies include anti-tissue transglutaminase antibodies (77.8%). End-stage liver disease markers like abdominal ascites (55.6%), variceal bleed (50.0%), acute or chronic kidney injury (16.7%), hepatocellular carcinoma (HCC) (11.1%), hepatic encephalopathy (HE) (50.0%), spontaneous bacterial peritonitis (SBP) (5.6%), and liver transplant (0.0%) were seen. The mean baseline MELD-Na score was 11.8, and albumin was 3.5 at the time of celiac diagnosis and mean MELD-Na was 11.8, and albumin was 3.5 six months after a gluten-free diet.

Conclusion

It is difficult to conclude any exact relationship between change in MELD-Na score after gluten-free diet, but an improving trend is noted in patients with higher MELD-Na score such as 17 or higher. There is no change or worsening of MELD-Na score in patients with lower MELD-Na score. There was no change in mean MELD-Na and albumin level after gluten-free diet.

## Introduction

Celiac disease (CD), also known as gluten-sensitive enteropathy or celiac sprue, is a small bowel disorder characterized by mucosal inflammation, villous atrophy, and crypt hyperplasia, which occurs upon exposure to dietary gluten and demonstrates improvement after the withdrawal of gluten from the diet [1, 2]. Although CD mainly affects small intestine injury resulting in malabsorption, more recently, it has been recognized to be a multisystem disorder that may affect other organs such as the skin, nervous system, heart, bone, and the liver [3-5]. The clinical symptoms of CD can vary from a classical malabsorption syndrome to more subtle atypical gastrointestinal (GI) manifestations or extraintestinal presentations like infertility, growth retardation, iron deficiency anemia, osteoporosis, and liver dysfunction [1, 6].

The liver disorder associated with Crohn's disease includes isolated liver enzyme elevation, autoimmune hepatitis, primary sclerosing cholangitis, non-alcoholic fatty liver disease and primary biliary cirrhosis [7-11]. Prevalence of elevated liver enzyme in adult population celiac disease is 40%, and data suggest that liver enzyme abnormalities are usually mild and respond to a gluten-free diet [2, 7, 12].

Cirrhosis affects up to 10% of the general population, and its prevalence is increasing [13]. Cirrhosis is a progressive disease associated with higher mortality and morbidity. It increases healthcare costs, so it is essential to recognize the celiac disease in the setting of cirrhosis and start the patient on a gluten-free diet to decrease healthcare costs by preventing office and hospital visits.

Studies about celiac and cirrhosis are not prevalent, so we conducted this study to evaluate a change in patient MELD-Na and albumin level from the time of celiac disease diagnosis to six months later, after implementing a gluten-free diet.

The model for end-stage liver disease (MELD) score is a prospectively developed and validated cirrhosis severity scoring system that uses a patient's laboratory values for serum bilirubin, serum creatinine, and the international normalized ratio (INR) to predict three-month survival. The MELD-Na score includes serum sodium as a factor in the calculation of the MELD score and is used by UNOS for prioritizing allocation of deceased donor livers for transplantation.

## Materials and methods

Methods

This retrospective study collected data of 18 patients with celiac disease and liver cirrhosis between January 1, 2006, and June 30, 2018. The institutional review board approved the study. The requirement of informed consent was waived at the time of approval due to the retrospective study. Patients were included in the study if they were above 18 years of age and had celiac disease and liver cirrhosis.

CD was diagnosed based on a celiac antibody and/or histopathological data. Celiac serology panel includes anti-tissue transglutaminase antibodies, anti-endomysial antibody, anti-gliadin antibody, and deamidated gliadin peptide.

The diagnosis of cirrhosis was made when these patients had two or more of the following three features: (1) imaging studies suggestive of cirrhosis (coarsening of echotexture, shrunken liver, nodular liver), (2) symptoms and/or signs of portal hypertension (esophageal varices or collaterals on ultrasonography), and (3) clinical and laboratory features consistent with liver cell dysfunction such as ascites, hypoalbuminemia, or hepatic encephalopathy. The severity of cirrhosis was assessed by calculating the MELD scores for all patients.

MELD-Na was calculated by capturing data like serum creatinine, bilirubin, INR, sodium, and dialysis at least twice in the past week. Additional variables like gender, ethnicity, serum IgA level, serum IgG level, human leukocyte antigen (HLA) type, and post-liver transplant mortality in 1.0 year were collected.

Patients diagnosed to have chronic liver disease (CLD) with CD were considered as cases. MELD-Na and albumin were calculated at the start of the gluten-free diet and six months later.

Statistical analysis

Descriptive statistics, including means and standard deviations or medians and interquartile ranges, were reported for continuous variables along with frequencies and percentages for categorical variables. Changes in MELD score and albumin lab values were calculated as the value at six months minus the value at baseline. Paired t-tests were used to describe a change in MELD-Na and albumin levels from the start of a gluten-free diet to six months later. The mean change, 95% confidence interval for mean change, and p-values are reported. Statistical analysis was completed with SAS 9.4 and p-values < 0.05 were considered statistically significant.

## Results

There were 18 patients identified as having both cirrhosis and celiac disease. The mean age at the time of celiac diagnosis was 53.6, 55.6% were male, and 94.4% were Caucasian. See Table 1 for a complete summary of patient characteristics.

**Table 1:** Summary of Patient Characteristics HLA: Human leukocyte antigen; IQR: Interquartile range.

n = 18 patients	n	%
Age at Celiac Diagnosis, mean (SD)	53.6 (10.2)	
Gender		
Female	8	44.4%
Male	10	55.6%
Ethnicity		
Asian	1	5.6%
Caucasian	17	94.4%
Histopathological Dx of Celiac		
Yes	8	44.4%
No	10	55.6%
Anti-tissue Transglutaminase Antibodies		
Yes	14	77.8%
No	4	22.2%
Anti-endomysial Antibody		
Yes	3	16.7%
No	15	83.3%
Anti-gliadin Antibody		
Yes	12	66.7%
No	6	33.3%
Deamidated Gliadin Peptide		
Yes	0	0.0%
No	18	100.0%
IgA Level, median (IQR) (n missing = 6)	359 (187, 593)	
HLA Testing		
Yes	0	0.0%
No	18	100.0%
Abdominal Ascites		
Yes	10	55.6%
No	8	44.4%
Variceal Bleed		
Yes	9	50.0%
No	9	50.0%
Acute or Chronic Kidney Injury		
Yes	3	16.7%
No	15	83.3%
Hepatocellular Carcinoma		
Yes	2	11.1%
No	16	88.9%
Hepatic Encephalopathy		
Yes	9	50.0%
No	9	50.0%
Spontaneous Bacterial Peritonitis		
Yes	1	5.6%
No	17	94.4%
Liver Transplant		
Yes	0	0.0%
No	18	100.0%

CD was diagnosed using celiac antibodies (100%) and histopathological data (44.4%). The most common celiac antibodies include anti-tissue transglutaminase antibodies (77.8%), followed by anti-gliadin antibody (66.7%) and anti-endomysial antibody (16.7%). HLA testing was not done in any patient. End-stage liver disease markers like abdominal ascites (55.6%), variceal bleed (50.0%), acute or chronic kidney injury (16.7%), hepatocellular carcinoma (HCC) (11.1%), hepatic encephalopathy (HE) (50.0%), spontaneous bacterial peritonitis (SBP) (5.6%), and liver transplant (0.0%) were seen.

The mean baseline MELD score was 11.8, and it was unchanged at six months after a gluten-free diet (mean change = 0.0, 95% CI for change: -2.4, 2.4, p = 1.0000). Mean albumin level at time of celiac diagnosis was 3.5 and it was also unchanged at six months (mean change = 0.1, 95% CI for change: -0.2, 0.3, p = 0.5282). Five patients (27.8%) showed an improvement (decrease) in the MELD score, three had no change (16.7%), and 10 (55.6%) had a deterioration (increase in score). Of the five patients with improvements in their MELD scores, the change ranged from a decrease of 11 points to a decrease of 4 points. Of the 10 patients with deterioration, the change ranged from an increase of 1 point to an increase of 8 points. The mean baseline MELD-Na was 17.8, 8.3, and 9.9 in patients with improvement, no change and deterioration in MELD-Na, respectively.

Seven patients (38.9%) had an increase in their albumin value, two (11.1%) had no change, and nine (50.0%) had a decrease. Among the seven patients with an increase in albumin, the change ranged from an increase of 0.1 to an increase of 1.2. Among the nine patients with a decrease in albumin, the decrease ranged from -0.4 to -0.1. The mean baseline albumin was 3.5, 3.8, and 3.3 in patients with improvement, no change and deterioration in albumin, respectively. See Tables 2-4 for results.

**Table 2:** MELD Score and Albumin at Baseline and Six Months After a Gluten-Free Diet MELD: Model for end-stage liver disease

n = 18 patients	n	%
MELD Score at Celiac Diagnosis, mean (S.D.)	11.8 (5.3)	
MELD Score at Six Months Post Gluten-Free Diet, mean (S.D.)	11.8 (4.5)	
Change in MELD Score mean (S.D.)	0.0 (4.9) 95% CI: (-2.4, 2.4) p = 1.0000	
Change in MELD Score		
Improvement (Decrease in score)	5	27.8%
No Change	3	16.7%
Decline (Increase in score)	10	55.6%
Albumin Level at Celiac Diagnosis	3.5 (0.6)	
Albumin Level After Celiac Prescription	3.5 (0.7)	
Change in Albumin Level mean (S.D.)	0.1 (0.4) 95% CI: (-0.2, 0.3) p = 0.5282	
Change in Albumin Level		
Albumin Value Increased	7	38.9%
No Change	2	11.1%
Albumin Value Decreased	9	50.0%

**Table 3:** Changes in MELD MELD: Model for end-stage liver disease

MELD Improvement	Baseline	6 Months	Change
1	25	14	-11
2	21	14	-7
3	17	12	-5
4	13	7	-6
5	13	9	-4
Baseline MELD, mean (S.D.)	17.8 (5.2)		
MELD No Change	Baseline	6 Months	Change
1	10	10	0
2	8	8	0
3	7	7	0
Baseline MELD, mean (S.D.)	8.3 (1.5)		
MELD Decline	Baseline	6 Months	Change
1	16	24	8
2	15	19	4
3	12	15	3
4	11	14	3
5	10	14	4
6	9	10	1
7	8	9	1
8	6	12	6
9	6	8	2
10	6	7	1
Baseline MELD, mean (S.D.)	9.9 (3.6)		

**Table 4:** Changes in Albumin

Albumin Increase	Baseline	6 Months	Change
1	3.8	4.1	0.3
2	3.7	3.8	0.1
3	3.7	3.8	0.1
4	3.6	4.1	0.5
5	3.5	3.8	0.3
6	3.3	4.5	1.2
7	3.0	3.9	0.9
Baseline Albumin, mean (S.D.)	3.5 (0.3)		
Albumin Increase	Baseline	6 Months	Change
Albumin No Change	Baseline	6 Months	Change
2	4.1	4.1	0.0
1	3.4	3.4	0.0
Baseline Albumin, mean (S.D.)	3.8 (0.5)		
Albumin Decrease	Baseline	6 Months	Change
1	4.5	4.1	-0.4
2	4.3	4.1	-0.2
3	3.5	3.3	-0.2
4	3.5	3.1	-0.4
5	3.4	3.2	-0.2
6	3.1	2.7	-0.4
7	3.1	3.0	-0.1
8	2.4	2.3	-0.1
9	2.3	2.1	-0.2
Baseline Albumin, mean (S.D.)	3.3 (0.7)		

## Discussion

Celiac disease is a multi-system disorder that may affect any organ. The prevalence of CD in patients with a CLD is reported to be 10-15 times higher compared to the general population [14, 15]. The liver can be involved in patients with celiac disease either due to coexistent autoimmune liver disease or coexistent alcohol, viral, metabolic, or nonalcoholic steatohepatitis, or it may remain cryptogenic [16-21].

The most common blood abnormalities in celiac disease include moderate elevation of transaminase, but alkaline phosphatase and bilirubin levels can also be elevated [20, 22, 23]. CD is present in about 9% to 10% of patients with chronic unexplained hypertransaminasemia [7, 24].

The mechanism of liver injury in CD is uncertain, but several pathophysiologic mechanisms are proposed such as increased intestinal permeability, mucosal damage, systemic autoimmunity, malnutrition, inflammation, and small intestinal bacterial overgrowth [25, 26]. The histologic findings in patients with CD are minimal Kupffer cell hyperplasia, macro-vesicular steatosis, and focal ductular proliferation [27].

The MELD is a valid measure of mortality risk in patients with end-stage liver disease [28]. The primary use of the MELD and MELD-Na scores is used as a disease severity index to help prioritize the allocation of organs for transplant.

In this research, we highlight the coexistence of cirrhosis with celiac disease and change in MELD-Na and albumin levels after a gluten-free diet. All patients had positive celiac serology, but only 44.4% had a histological diagnosis. The sample size is too small to statistically compare baseline MELD-Na and albumin changes.

It is difficult to conclude any exact relationship between change in MELD-Na score after gluten-free diet, but an improving trend is noted in patients with higher MELD-Na score such as 17 or higher. There is no change or worsening of MELD-Na score in patients with lower MELD-Na score. It is difficult to draw any specific trend with albumin.

Several studies have shown a delay in the progression of liver disease and improvement of nutritional status after a gluten-free diet [29]. Few studies proved delays in liver transplantation and removal from transplant list due to improvement after gluten-free diet (GFD) [20].

There are several limitations of our study, such as 1) etiology of cirrhosis was not collected, 2) small sample size and follow-up period, 3) whether patients were on cirrhosis treatment or not.

## Conclusions

We highlight the coexistence of cirrhosis with celiac disease and change in MELD-Na and albumin levels after a gluten-free diet. It is difficult to conclude any exact relationship between change in MELD-Na score after gluten-free diet, but an improving trend is noted in patients with higher MELD-Na score such as 17 or higher. There is no change or worsening of MELD-Na score in patients with lower MELD-Na score. It is difficult to draw any specific trend with albumin as sample size is too small. Larger studies and a longer follow-up are needed to determine whether improvement seen in celiac disease patients is persistent and whether the cirrhosis will either progress or regress.
